# Assessment of the domains of quality of life in the geriatric population

**DOI:** 10.4103/0019-5545.55940

**Published:** 2005

**Authors:** Ankur Barua, R. Mangesh, H.N. Harsha Kumar, Mathew Saajan

**Affiliations:** * Assistant Professor, Department of Community Medicine, Kasturba Medical College, Manipal; ** Assistant Professor, Department of Community Medicine, Kasturba Medical College, Manipal; *** Postgraduate Student, Department of Community Medicine, Kasturba Medical College, Manipal; **** Intern, Department of Community Medicine, Kasturba Medical College, Manipal

**Keywords:** Quality of life, geriatric population, domains, well-being

## Abstract

**Background:**

The number of studies from India on geriatric health problems, particularly mental health disorders and quality of life, is limited.

**Aim:**

This cross-sectional study was carried out in the geriatric population visiting the Dr T.M.A. Pai Rotary Hospital, Karkala, Karnataka. It examined the different domains of quality of life according to sociodemographic variables.

**Methods:**

We studied 70 individuals in the geriatric age group (≥60 years) who visited the hospital over a period of two months from March 2003 to April 2003. The results were expressed in terms of mean and SE of mean. Z-test and one-way ANOVA test were applied to compare the mean scores of different variables under the four domains. A p value <0.05 was considered significant.

**Results:**

The total mean score, as well as the mean scores in each of the four domains studied were similar in men and women as well as literates and illiterates. The mean scores of subjects in various age groups differed significantly in the domains of physical, psychological and social relations, while single and married subjects differed significantly in the domains of environmental and social relations. Overall well-being was significantly affected in those who were single (unmarried/widowed) or in the age group of 60–69 years.

## INTRODUCTION

The effects of disease and health interventions on an individual's quality of life can be measured by quality of life assessments. All the aspects of ‘health status’, ‘lifestyle’, ‘life satisfaction’, ‘mental state’ and ‘well-being’ together reflect the multidimensional nature of quality of life.^1^,^2^ However, in India, only a few studies have explored geriatric health problems, particularly mental health disorders and quality of life.^3^–^5^ Considering this background, this mental health study was conducted to examine the different domains of quality of life affected by sociodemographic factors in the geriatric population visiting the Dr T.M.A. Pai Rotary Hospital, Karkala, Karnataka.

## METHODS

This cross-sectional study was carried out in 70 individuals of the geriatric age group (≥60 years of age) visiting the Dr T.M.A. Pai Rotary Hospital, Karkala, Karnataka over two months from March 2003 to April 2003.

### Study instrument

A face sheet containing information on the household of the respondent was used for data collection. We used the World Health Organization-Quality of life (WHOQOL)-BREF^6^,^7^ instrument to assess the quality of life. The WHOQOL-BREF instrument was translated into Kannada by researchers and back-translated into English by another expert not acquainted with the original versions. The back-translated version was subsequently compared with the original by a psychiatrist for conceptual equivalence of the items. The translated version of this instrument was pre-tested on a subsample before it was used on the study population to ensure feasibility and acceptability.

### Data collection

No sampling procedure was applied to select our study subjects. Individuals ≥60 years of age, who consented to give the interview, were included in the study. The interviews were conducted in the hospital. Each interview took approximately 30 minutes. After initial introduction and rapport building, the sociodemographic data were collected, following which the respondents were administered the WHOQOL-BREF.

### Data analysis

Data were tabulated and analysed using the Statistical Package for Social Sciences (SPSS) version 7.5. The results were expressed in terms of mean and SE of mean. Z-test and one-way ANOVA test were applied to compare the mean scores of different variables under the four domains. A p value <0.05 was considered significant.

The majority of the study population belonged to the age group of 60–69 years (80%). Sixty per cent of the study population were females and 75.7% literate. While 78.6% of the individuals were married, 21.4% were either unmarried or widowed. The mean age of the study population was 65.8 years (SD±4.9).

## RESULTS AND DISCUSSION

Table 1 compares the mean scores in physical, psychological, social relations and environmental domain according to sex, age group, marital status and literacy status.

**Table 1:** Comparison of sociodemographic variables and mean scores of all domains

	Mean score of domains (SE)									
	
Sociodemographic variable	Physical		Psychological		Social relations		Environmental		Total	
Sex											
Female	54.9	(2.9)	52.0	(2.6)	56.9	(3.5)	52.9	(2.7)	216.7	(10.2)
Male	51.2	(3.6)	51.3	(2.5)	55.9	(2.7)	57.1	(3.2)	215.6	(8.6)
p	0.435		0.857		0.840		0.336		0.939	
Age group (years)									
60–69	40.5	(3.7)	39.5	(3.4)	45.2	(4.6)	53.0	(5.0)	178.3	(11.5)
≥70	56.7	(2.5)	54.7	(1.9)	59.3	(2.6)	55.0	(2.3)	225.7	(7.8)
p	0.004*		0.001*		0.016*		0.722		0.006*	
Marital status									
Single	51.5	(5.0)	45.0	(4.4)	42.2	(6.6)	44.2	(5.0)	182.7	(17.9)
Married	54.0	(2.5)	53.4	(2.0)	60.0	(2.2)	57.2	(2.1)	224.6	(7.2)
p	0.674		0.064		0.002*		0.012*		0.016*	
Literacy status									
Illiterate	51.6	(3.9)	52.6	(3.9)	58.3	(4.7)	54.6	(4.4)	217.0	(13.6)
Literate	54.0	(2.7)	51.4	(2.1)	55.9	(2.8)	54.5	(2.4)	215.9	(8.2)
p	0.648		0.778		0.662		0.993		0.994	

* A p value <0.05 is significant.

1. The total mean score as well as the mean scores in each of the four domains for both men and women were found to be similar. The difference between the two groups was not found to be statistically significant for any of the four domains.
2. The mean scores of the two age groups of 60–69 years and ≥70 years were found to differ significantly in the domains of physical (p=0.004), psychological (p=0.001) and social relations (p=0.016). This difference between the two groups was also found to be statistically significant for the total mean score in all the domains (p=0.006). Hence, the overall well-being was significantly affected for those who were in the age group of 60-69 years.
3. The mean scores of those single and married differed significantly in the domains of environmental (p=0.012) and social relations (p=0.002). This difference between the two groups was also found to be statistically significant for the total mean score of all the domains (p=0.016). Thus, the overall well-being was significantly affected for those who were single (unmarried and widowed).
4. The total mean score, as well as the mean scores in each of the four domains for both literate and illiterate subjects were found to be similar. A little difference between the two groups was, however, not found to be statistically significant for any of these four domains.

### ANOVA test results

One-way ANOVA test was applied to compare the mean scores of all the domains and the total score for each of the four independent variables, i.e. sex, age group, marital status and literacy status.

#### Sex

No significant difference in the mean scores was observed in the physical (p=0.435), psychological (p=0.857), social (p=0.840) and environmental (p=0.336) domains, and also on total scores (p=0.939) among men and women.

#### Age group

There was a significant difference in the mean scores in the physical (p=0.004), psychological (p=0.001) and social (p=0.016) domains, and also on total scores (p=0.006) among those in the age groups of ≥60–69 years and ≥70 years.

#### Marital status

There was a significant difference in the mean scores in the social (p=0.002) and environmental (p=0.012) domains, and also on total scores (p=0.016) among single and married subjects.

#### Literacy status

There was no significant difference in the mean scores in the physical (p=0.648), psychological (p=0.778), social (p=0.662) and environmental (p=0.993) domains, and also on total scores (p=0.944) among illiterate and literate subjects.

## CONCLUSION

The total mean score, as well as the mean scores in each of the four domains were similar in men and women as well as among literate and illiterate subjects. The mean scores of those in the 60–69 and ≥70 years’ age groups differed significantly in the domains of physical, psychological and social relations, while single and married subjects differed significantly in the domains of environmental and social relations. The overall well-being was significantly affected for those who were single (unmarried/widowed) or in the age group of 60–69 years.
